# Identification of SKOR2 IgG as a novel biomarker of paraneoplastic neurologic syndrome

**DOI:** 10.3389/fimmu.2023.1243946

**Published:** 2023-09-18

**Authors:** Mohamed Rezk, Sean J. Pittock, Ronak K. Kapadia, Andrew M. Knight, Yong Guo, Pranjal Gupta, Reghann G. LaFrance-Corey, Anastasia Zekeridou, Andrew McKeon, Surendra Dasari, John R. Mills, Divyanshu Dubey

**Affiliations:** ^1^ Department of Neurology, Mayo Clinic, Rochester, MN, United States; ^2^ Department of Laboratory Medicine and Pathology, Mayo Clinic, Rochester, MN, United States; ^3^ Center for Multiple Sclerosis and Autoimmune Neurology, Mayo Clinic, Rochester, MN, United States; ^4^ Department of Clinical Neurosciences, Cumming School of Medicine, University of Calgary, Calgary, AB, Canada

**Keywords:** paraneoplastic neurologic syndrome, paraneoplastic encephalitis, antibody biomarker, gall bladder adenocarcinoma, lung adecarcinoma

## Abstract

**Introduction:**

The development of new autoantigen discovery techniques, like programmable phage immunoprecipitation sequencing (PhIP-Seq), has accelerated the discovery of neural-specific autoantibodies. Herein, we report the identification of a novel biomarker for paraneoplastic neurologic syndrome (PNS), Sloan-Kettering-Virus-Family-Transcriptional-Corepressor-2 (SKOR2)-IgG, utilizing PhIP-Seq. We have also performed a thorough clinical validation using normal, healthy, and disease/cancer control samples.

**Methods:**

Stored samples with unclassified staining at the junction of the Purkinje cell and the granule cell layers were analyzed by PhIP-Seq for putative autoantigen identification. The autoantigen was confirmed by recombinant antigen-expressing cell-based assay (CBA), Western blotting, and tissue immunofluorescence assay colocalization.

**Results:**

PhIP-Seq data revealed SKOR2 as the candidate autoantigen. The target antigen was confirmed by a recombinant SKOR-2-expressing, and cell lysate Western blot. Furthermore, IgG from both patient samples colocalized with a commercial SKOR2–specific IgG on cryosections of the mouse brain. Both SKOR2 IgG-positive patients had central nervous system involvement, one presenting with encephalitis and seizures (Patient 1) and the other with cognitive dysfunction, spastic ataxia, dysarthria, dysphagia, and pseudobulbar affect (Patient 2). They had a refractory progressive course and were diagnosed with adenocarcinoma (Patient 1: lung, Patient 2: gallbladder). Sera from adenocarcinoma patients without PNS (n=30) tested for SKOR2-IgG were negative.

**Discussion:**

SKOR2 IgG represents a novel biomarker for PNS associated with adenocarcinoma. Identification of additional SKOR2 IgG-positive cases will help categorize the associated neurological phenotype and the risk of underlying malignancy.

## Introduction

Paraneoplastic neurologic syndromes (PNS) are a group of disorders affecting one or more levels of the neuroaxis associated with underlying tumors (1). An antibody- or autoantigen-specific cell-mediated immune response against neural antigens expressed in the tumor is the potential etiology for these rare but refractory disorders (2). In recent years, a wide variety of neurological presentations and autoantibodies have been associated with PNS (1). The development of novel autoantigen discovery techniques, such as programmable phage immunoprecipitation sequencing (PhIP-Seq), has led to the identification of putative autoantigens such as Kelch-like protein 11 (KLHL11) and cavin-4 (caveolae-associated protein 4) (3–5).

Here we report the discovery of novel autoantibodies targeting the Sloan-Kettering Virus Family Transcriptional Corepressor 2 (SKOR2) protein, present in both serum and cerebrospinal fluid (CSF) of two patients diagnosed with CNS autoimmunity and an underlying malignancy.

## Methods

### Standard protocol approvals, registrations, and patient consent

This study was approved by the Mayo Clinic Institutional Review Board (IRB) under number (08-00647). The IRB was approved under the criteria for waiver of informed consent [45 CFR 46.116(d)]. The relevant IRB was further approved in consideration of HIPAA waiver criteria [45 CFR Part 229 164 - Security and Privacy Rule, Subpart E].

### Patient selection

Between 2007 and 2020, we collected 41 archived specimens (30 sera and 11 cerebrospinal fluids [CSFs]) that showed a nuclear/cytosolic staining pattern at the junction of the Purkinje cell layer and the granule layer of the cerebellum when tested by an indirect immunofluorescence assay (IFA) on cryosectioned mouse tissues. Of these, two exhibited identical staining and were used for this project.

### Phage immunoprecipitation sequencing

The samples were incubated with 10^10^ plaque-forming units per milliliter of the whole human proteome phage-display library (5). The phage particles were incubated with serum (1:5000) or CSF (1:100) overnight at 4°C and isolated using magnetic protein G beads (Invitrogen). Captured phage particles were extensively washed in immunoprecipitation wash buffer, and the embedded DNA-coded fragments were amplified by polymerase chain reaction (PCR). Amplified PCR products were prepared for next-generation sequencing with the Illumina TruSeq Nano DNA kit and sequenced with the MiSeq platform. Sequenced reads were processed using a previously described (5) in-house-developed bioinformatics pipeline to identify the putative autoantigen.

### Confirmation and verification of the putative autoantigen

The putative autoantigen was confirmed by testing the two patients’ sera and/or CSF with identical tissue immunofluorescence staining patterns using a protein expression vector-transfected COS7 cell-based assay (CBA), Western blotting, and IFA colocalization on cryosectioned mouse brain. A thorough clinical review of the two patients’ charts was performed. We also tested the remaining 39 samples of the 41 archived specimens demonstrating a nuclear/cytosolic staining pattern at the junction of the Purkinje cell layer and the granule layer of the cerebellum using CBA.

To test the autoantigen specificity, serum samples from patients with lung adenocarcinoma without neurological syndromes (n=30), neuromyelitis optica spectrum disorder (NMOSD) (n=19), Sjogren’s syndrome (n=10), systemic lupus erythematosus (n=10), Purkinje cell cytoplasmic antibody type 1 (PCA1, anti-Yo) positives with breast/ovarian/uterine adenocarcinomas (n=16), healthy controls (n=60), CSF samples from multiple sclerosis (MS) (n=12), and normal pressure hydrocephalus (NPH) (n=10) were tested using CBA.

Additionally, tissue IFA specificity was evaluated by testing sera from patients with NMOSD (n=40), systemic lupus erythematosus (n=30), PCA1 IgG positive patients (n=9), anti-neuronal nuclear antibody type 1 (ANNA1, anti-Hu) IgG positive patients (n=20), and healthy controls (n=120). CSF samples from multiple sclerosis (MS) (n=50) and normal pressure hydrocephalus (NPH) (n=25) were also tested. 

### Tissue immunofluorescence assay

Patient serum and commercial rabbit antibodies were tested on a cryosectioned composite of murine tissue (brain, kidney, and gut mucosa; Scimedx Corporation, Denville, USA). Sections were fixed with 4% paraformaldehyde for 1 min, then permeabilized with 3-([3-cholamidopropyl] dimethylammonio)-1-propane sulfonate, 0.5%, in PBS for 1 min, and then blocked for 1 h with normal goat serum (10% in PBS). After PBS rinsing, sera and CSF patient specimens (serum preabsorbed at 1:240 dilution with bovine liver powder; CSF non-absorbed at 1:10 dilution) were applied and incubated for 40 min. This was followed by another PBS wash and a 30-min incubation with a human IgG-specific secondary antibody (1:2000) conjugated with FITC (Southern Biotechnology) in 10% goat serum PBS. After washing in PBS, coverslips were applied with ProLong Gold antifade mounting medium containing DAPI (Molecular Probes, Thermo Fisher Scientific, Waltham, USA). For dual staining on murine tissue, we applied patient serum (serum preabsorbed at 1:480 dilution with bovine liver powder) or CSF (CSF non-absorbed at 1:20 dilution) and rabbit polyclonal SKOR2–specific IgG (1:300, Novus Biologicals, Centennial, USA) and secondary antibodies (1:200, tetramethylrhodamine-conjugated goat anti–rabbit IgG and goat anti–Human IgG, Southern Biotechnology, Birmingham, USA). Confocal images were captured using an LSM710 microscope (63, 40, or 20 × water immersion lens; Carl Zeiss Inc., Oberkochen, Germany).

### Cell-based assay

COS7 cells were transfected with plasma encoding full-length GFP-tagged human SKOR2 (vector, pcDNA3.1 (+)-C eGFP plasmid, Genscript, Piscataway, USA). After transfection, COS7 cells were incubated for 16-24 h (at 37°C in a humidified atmosphere of 95% air and 5% CO2). COS7 cells were then fixed (4% paraformaldehyde, 15 min), permeabilized (0.2% Triton X-100, 10 min), and blocked with 10% normal goat serum in PBS for 1 h. After PBS washing, cells were incubated with patient serum (1:200 dilution) or rabbit SKOR2-specific IgG (1:300 dilution) for 40 min. After PBS washing and incubation with secondary antibodies (1:200 tetramethylrhodamine [TRITC]-conjugated goat anti-rabbit IgG and goat anti-Human IgG; Southern Biotech, Birmingham, USA). Coverslips were mounted using ProLong Gold antifade medium (containing 4,6- diamidino-2-phenylindole [DAPI]; Molecular Probes, Thermo Fisher Scientific, Waltham, USA). Assays were graded by at least two independent reviewers.

### Western blot

Cells were washed once in cold 1X PBS, and 1 mL of cold RIPA (50 mM Tris HCL pH 7.5, 150 mM NaCL, 1.0% Triton-X, 0.1% SDS, Roche protease inhibitor tablet, Roche Life Science, Sigma Aldrich, St. Louis, USA) was added 40 h after transfection with the SKOR2-GFP plasmid. Cells were scraped off the dish, and lysates were homogenized and allowed to lyse in RIPA for an additional 30 min at 4°C before further homogenization. The lysate was used for Western blotting (separated in a 4-15% polyacrylamide gel, Criterion Bio-Rad, Bio-Rad Laboratories, Hercules, USA), transblotted to nitrocellulose membranes (Roche Life Science, Sigma Aldrich), and blocked in 10% milk powder in Tris-buffered saline and Tween 20 (TBST, Sigma Aldrich). Individual lanes were probed with sera from healthy controls (1:100), candidate patients (1:100), or commercial SKOR2-specific IgG (1:1000). The nitrocellulose membrane was then washed with TBST and probed with anti-Human HRP or anti-rabbit HRP (Promega, Madison, USA). Blots were visualized with SuperSignal West Pico PLUS (Thermo Scientific) and Azure Biosystems (Azure Biosystems, Inc., Dublin, USA) instrumentation.

### Immunohistochemistry

Formalin-fixed, paraffin-embedded, 5 µm-thick sections were stained with hematoxylin and eosin (H&E). Immunohistochemistry was performed with the EnVisionTM FLEX immunohistochemistry system (DAKO) (Agilent Technologies, Santa Clara, USA) after steam antigen retrieval with citric acid buffer (pH 6.0). Primary antibodies against SKOR2 (1:500, #NBP2-12565, Novus Biologicals) were incubated at 4°C overnight. Histopathology was reviewed and photographed with a brightfield microscope (Olympus BX53, Olympus Corporation, Tokyo, Japan).

## Results

### Discovery of the novel autoantigen

**Figure 1:**
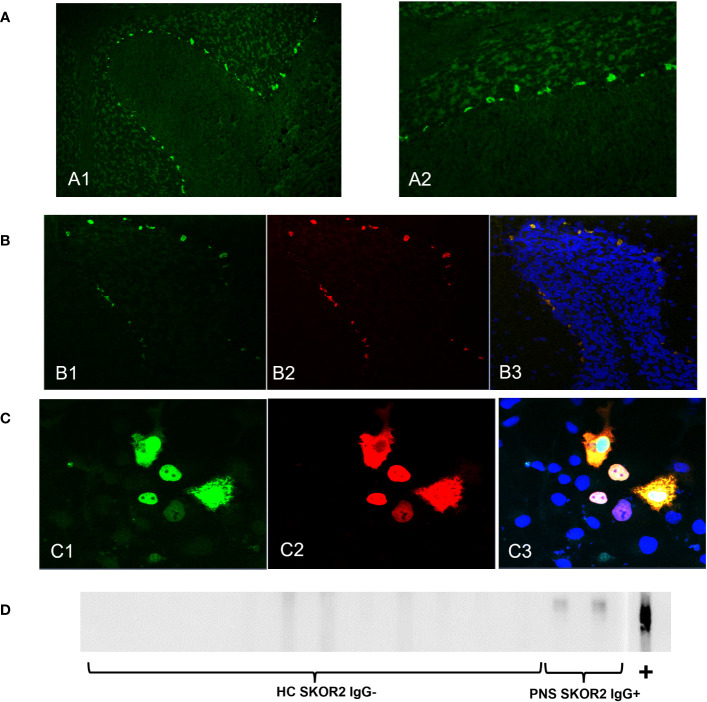
SKOR2 IgG identification and confirmation. **(A)** Serum IgG from Patient 2 demonstrates nuclear staining at the junction of the Purkinje cell layer and the granule layer of the cerebellum in indirect tissue immunofluorescence on cryosectioned mouse cerebellum (20x magnification, (A1) and 40x magnification, (A2)). **(B)**. Dual immunostaining of cryosectioned mouse brain tissue with commercial SKOR2–specific rabbit IgG in green (B1) and with patient IgG (CSF from Patient 1) in red (B2) demonstrated colocalization; nuclei are stained blue by 4′,6-diamidino-2-phenylindole; the merged image is in yellow (B3). **(C)** Tetramethylrhodamine-conjugated anti–Human IgG (serum from Patient 1) in red (C2) binds to recombinant SKOR2 protein–tagged expression in the transiently transfected COS7 cells in green (C1) the merged image is in yellow with nuclei in blue (C3). **(D)** Western blot of COS7 cell lysate containing recombinant SKOR2 demonstrated binding of IgG in sera from both paraneoplastic neurologic syndrome cases (indicated as PNS SKOR2 IgG+) and commercial SKOR2 antibodies (indicated as +) to an approximately 130-kD protein; no IgG bound to the serum of healthy control individual (indicated as HC SKOR2 IgG-).

A unique nuclear murine tissue IFA staining pattern restricted to the junction of the Purkinje cell layer and the granule layer, with faint punctate staining in the brainstem and diencephalon, was identified in samples from two patients (Patient 1, serum [titer≥1:1920] and CSF [titer≥1:100]; Patient 2, serum [titer≥1:1920]) (**Figure 1A**). No staining was observed in the molecular layer of the cerebellum, hippocampus, cortex, gastric smooth muscle, gastric mucosa, or kidney. Serum and CSF from each of the two patients were tested by whole-human proteome PhIP-Seq. Bioinformatic analysis of the PhIP-Seq data revealed SKOR2 as the candidate antigen (**Supplementary material**).

*Validation of the novel autoantigen*

Patient IgG from these specimens was also colocalized with a commercial SKOR2–specific IgG on cryosections of mouse brain tissue by immunostaining (**Figure 1B**). Both patient samples tested positive for CBA using SKOR2–transfected COS7 cells (**Figure 1C**). Furthermore, patient sera produced a positive band on a Western blot on lysate containing full-length SKOR2 protein (**Figure 1D**).

### Specificity of SKOR2 IgG

All healthy and disease-control sera and/or CSF tested on IFA were negative. However, on CBA, one case of aquaporin-4-positive neuromyelitis optica was positive (all remaining NMOSD sera and other healthy or disease-control sera and CSF were negative on CBA). All 39 samples with a similar IFA staining pattern that was not completely consistent with the last two patients were negative on CBA. Consequently, we tested this sample by tissue indirect immunofluorescence assay, which did not reveal the unique staining pattern or colocalize with a commercial SKOR2–specific IgG. The sample was also tested by SKOR2 overexpression lysate Western blot and was found to be negative. Additionally, whole human proteome PhIP-Seq did not show a high SKOR2 enrichment score, suggesting a false-positive SKOR2 CBA result.

### Case presentations

SKOR2 IgG-positive patients had subacute or chronic progressive central nervous system involvement, one presenting with encephalitis and seizures (Case 1) and the other with spastic ataxia, dysarthria, dysphagia, cognitive dysfunction, and pseudobulbar affect (Case 2). Both patients were diagnosed with adenocarcinomas of the lung and gallbladder, respectively.

#### Case 1

**Figure 2:**
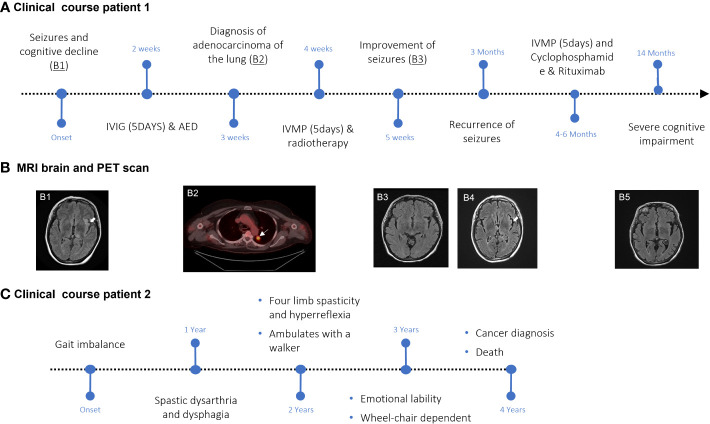
Disease course and clinical studies in SKOR2 IgG-positive patients. **(A)** Graphic time course of events for Case 1. Major events are indicated in black. **(B)** FLAIR sequence of the MRI shows hyperintensities in the left external capsule, insula, and thalamus ((B1), arrowhead). The hypermetabolic lesion in the left upper lobe (arrow) was confirmed by biopsy to be lung adenocarcinoma (B2). Post-immunotherapy MRI shows the resolution of findings observed on the first MRI ((B3), arrowhead). A recurrence of the left insular hyperintensity can be seen (B4). The last follow-up MRI demonstrated global volume loss (B5). **(C)** Graphic time course of events for Case 2. Major events are indicated in black.

A 64-year-old woman was admitted to the hospital after presenting with multiple episodes of sensorimotor focal seizures of the right arm and leg with impaired awareness over a two-week period. On two occasions, these focal seizures progressed to bilateral tonic-clonic seizures. The patient was noted to have had a decline in cognition (specifically short-term memory) for six months prior to hospitalization (**Figure 2A**). At the time of admission, a MRI of the brain showed T2/FLAIR hyperintensities in the left thalamus, external capsule, insula, and temporoparietal cortex (**Figure 2**B1). Additionally, there were subtle T2/FLAIR hyperintensities in the bilateral medial temporal lobes. Focal epileptiform discharges along with periodic lateralized discharges were seen in the left temporal lobe. There was also mild slowing in the same areas. CSF analysis revealed normal protein and cell counts. 14-3-3 protein in the CSF was negative. No malignant cells were observed on CSF cytology. A 2.1-cm nodule in the upper lobe of the left lung was seen on chest CT. A positron emission tomography (PET) scan of the whole body showed that the nodule was intensely hypermetabolic and suggestive of malignancy (**Figure 2**B2), which was confirmed by biopsy to be invasive pulmonary adenocarcinoma. The tumor was considered inoperable due to the patient’s poor condition. The patient was started on radiation therapy (6000 cGy in eight fractions). The patient was also started on four anticonvulsant medications with limited improvement in clinical and electrographic seizures. For the management of refractory seizures, intravenous immunoglobulin (IVIG) at 2g/kg for 5 days was administered, followed by intravenous methylprednisolone (IVMP) at 1 gram daily for 5 days during the first 3 weeks of hospitalization. Subsequent imaging performed 4 weeks after treatment showed near complete resolution of T2/FLAIR hyperintensities, although the patient remained severely cognitively impaired (**Figure 2**B3). After being off immunotherapy for 2 months, the patient had a clinical relapse in the form of worsening cognitive deficits and recurrence of seizures, with MRI brain imaging showing recrudescence of previous findings (**Figure 2**B4). She was treated with cyclophosphamide (750mg/m2 monthly for 6 months – stopped after two treatments due to drug sensitivity) after a repeat course of 1g IVMP over 5 days, which contributed to some clinical improvement. She then received two induction doses of rituximab 1(000 mg IV) separated by 14 days approximately 6 months following her initial admission to the hospital. This was followed by two maintenance doses of rituximab (500 mg IV) every 6 months after induction. Follow-up MRI brain imaging 10 months after hospitalization showed mild residual signal abnormalities of the left thalamus and insular cortex with the development of global cerebral volume loss (**Figure 2**B5). The patient was found to have persistent severe cognitive impairment on clinical follow-up 14 months after initial hospital admission. The patient is currently residing in a long-term care facility.

#### Case 2

A 70-year-old woman presented with complaints of gait imbalance (**Figure 2C**). One year after the onset of gait dysfunction, she also developed dysarthria. Her examination revealed spastic dysarthria and pyramidal dysfunction in the four extremities in the form of spasticity and hyperreflexia, in addition to bilateral Babinski signs. She had a markedly slow alternating motion rate of the tongue, hands, and legs. Her gait was wide-based, ataxic, and spastic. The sensory examination was normal.

Brain, cervical, and thoracic spine MRIs were unremarkable. Electrodiagnostic studies revealed long-duration, high-amplitude, polyphasic motor unit potentials, suggestive of a neurogenic process. No spontaneous insertional activity (fibrillations or fasciculations) was observed in any of the muscles tested. A comprehensive evaluation for myeloneuropathy etiologies, which included vitamin B12 level, folic acid level, human T-cell lymphotropic virus type 1/type 2 antibody, human immunodeficiency virus antigen, genetic testing for spinocerebellar ataxias, Friedreich’s ataxia, and hereditary spastic paraplegia, was negative. No cancer workup was performed during this initial evaluation. Over the next 2 years, the patient deteriorated and developed cognitive dysfunction, emotional lability, and dysphagia. She was subsequently found to have developed a widely metastatic gallbladder carcinoma, which was not amenable to surgery, and she died. No autopsy was performed.

*SKOR2 immunoreactivity in tumor tissue*

**Figure 3:**
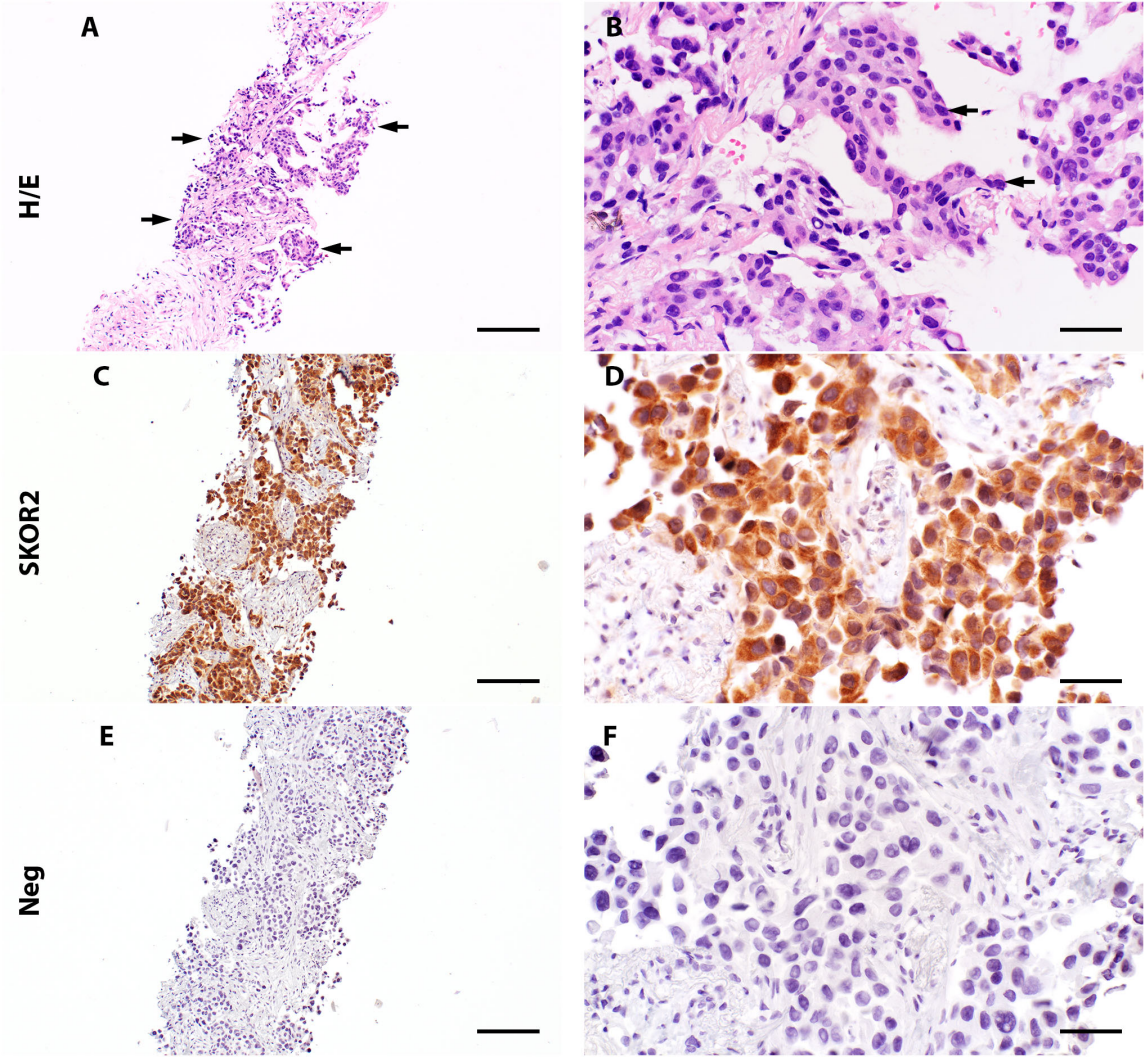
Expression of SKOR2 in pulmonary adenocarcinoma. **(A, B)** A lung biopsy from patient 1 (Diagnosed with pulmonary adenocarcinoma) shows neoplastic cells (indicated with arrows) with a high nuclear/cytoplasmic (N/C) ratio (H/E staining, high-power image in **(B)**). **(C, D)** Immunohistochemistry reveals variable cytoplasmic SKOR2 immunoreactivities in the neoplastic cells. **(E, F)** A negative control by omitting the primary antibody shows no signal in the following section. Scale bars: 200 μm **(A, C, E)**; 50 μm **(B, D, F)**.

Histopathologic assessment of the lung adenocarcinoma tissue (Case 1) from a single seropositive patient revealed cytoplasmic and nuclear SKOR2 immunoreactivity in the tumor cells (**Figure 3**).

## Discussion

We report the discovery and validation of autoantibodies against the SKOR2 protein, a novel biomarker for PNS, in two patients with progressive neurological syndromes and an underlying tumor. We found this autoantigen in a cohort of patients whose sera and/or CSF exhibited a distinct nuclear staining pattern, predominantly at the junction of the Purkinje cell layer and granule layer of the cerebellum on murine brain tissue IFA. Additionally, we demonstrated the expression of SKOR2 in lung adenocarcinoma tissue, supporting that the SKOR2 autoantigen may be pathogenically associated with the PNS.

Both patients with SKOR2 IgG seropositivity had CNS involvement. The first patient presented with encephalitis in the form of refractory seizures and subacute, progressive cognitive decline. The seizures initially responded favorably to immunotherapy but relapsed after being off immunosuppression for 2 months. This patient’s overall long-term outcome was poor despite the use of second-line immunosuppressive agents. The second patient developed progressive pyramidal dysfunction with gait ataxia. The presentation consisted of multifocal progressive upper motor neuron dysfunction along with brainstem/cerebral involvement. Notably, the multifocal neurologic symptoms, such as encephalomyelitis, were characterized as a high-risk paraneoplastic phenotype (6). Such phenotypes have been reported with high-risk paraneoplastic autoantibodies, such as ANNA1 (anti-Hu) (7) and collapsin response-mediator protein-5 (CRMP5, anti-CV-2) (8). Furthermore, EMG in Case 2 showed changes suggestive of coexisting anterior horn cell involvement, which has been reported in association with various autoimmune or paraneoplastic autoantibodies such as IgLON5 IgG (9) or Ma2 IgG (10). Both cases had a refractory and progressive clinical course. A similar refractory course was reported in PNSs associated with other onconeural or high-risk paraneoplastic autoantibodies targeting intracellular autoantigens such as PCA1 or KLHL11 (11, 12).

Small cell lung cancer (SCLC) is the most common histologic type of lung cancer associated with PNS, commonly in the setting of ANNA1 IgG seropositivity in the form of encephalomyelitis/sensory neuronopathy (13, 14). Lung or gallbladder adenocarcinomas, on the other hand, are not as commonly associated with PNS (1, 2) (13). However, there are some reports of PCA2 (microtubule-associated protein 1B) (15), ANNA2 (anti-Ri) (16), Ma2 (17), or ANNA1IgG (7) seropositivity with non-small cell lung cancer that include adenocarcinoma, even though these associations are not as well characterized as ANNA1 IgG and SCLC (18) or KLHL11 IgG and testicular germ cell tumor (3, 12). Reports of PNS cases associated with lung or gallbladder adenocarcinomas are summarized in **Table 1** (33–35). Limited paraneoplastic autoantibody biomarkers in patients presenting with progressive neurological syndrome in the setting of lung or gallbladder adenocarcinomas make the diagnosis of PNS challenging. The cases presented in this study add to the growing spectrum of PNS, not only in terms of describing novel autoantibodies but also in terms of organ and histologic cancer types triggering paraneoplastic autoimmunity.

**Table 1:** Summary of paraneoplastic neurologic syndromes reported with adenocarcinoma of the lung or gallbladder.

Age at diagnosis/sex	Initial symptoms	Adenocarcinoma organ	PNS phenotype	Autoantibody results	Time from symptom onset to cancer diagnosis	Outcome
64/F	Focal seizures with impaired awareness	Lung	Encephalitis	SKOR2	6 months	Severe cognitive impairment
77/F (19)	Gait instability and dysarthria	Lung	Brain stem encephalitis	Ma2	2 months	Died 7 months after onset of neurological symptoms
55/M (20)	Cognitive and functional decline	Lung	Limbic encephalitis	Ma2	4 months	Died of septic shock
43/F (21)	Limb paresthesia and stiffness	Lung	Stiff person syndrome	ANNA2	9 months	Stabilized
72/M	Cognitive dysfunction/encephalopathy	Lung	Encephalitis	AMPH	Not mentioned	Died after transient improvement with steroids
67/F (22)	Painful lower limb muscle spasms	Lung	Stiff person syndrome	GAD65	2 years	Improved with immunotherapy
75/M (23)	Bilateral lower limb weakness and gait disturbance	Lung	Lambert-Eaton myasthenic syndrome	P/Q-type VGCC	Not mentioned	Improved with tumor resection
72/M (24)	Seizures and left hemichorea	Lung	Encephalitis	Caspr2	1 month	Not mentioned
65/M (25)	Bilateral lower limb pain and tingling sensation	Lung	Mononeuritis multiplex	Not tested	2 months	Not mentioned
63/F (26)	Subacute progressive cognitive decline	Lung	Limbic encephalitis	ANNA1	6 months	Palliative care due to extensive cancer spread
55/M (27)	Vomiting and abdominal distension	Lung	Intestinal pseudo-obstruction	ANNA1	2 months	Not mentioned
80/M (28)	Progressive ascending muscle weakness in the legs and arms	Lung	Polyradiculoneuropathy	GM1	Not mentioned	Temporary improvement with corticosteroid until relapse 6 months later, followed by progressive course.
58/M (29)	Rapidly progressive memory decline and myoclonic jerks	Lung	Limbic encephalitis	Not tested	2 weeks	Improved with immunotherapy and tumor resection
Young/F (30)	Spastic quadriparesis, dysarthria, and bulbar symptoms	Lung	Primary lateral sclerosis	Negative	9 months*	Died 6 months after onset of neurological symptoms
66/F (31)	Right hemiballismus and dysarthria	Lung	Encephalopathy	Negative	4 months*	Died despite immunotherapy
70/F	Gait imbalance and dysarthria	Gallbladder	Encephalomyelitis	SKOR2	4 years	Died
80/F (32)	Paresthesia with glove and stocking distribution with bilateral lower limb weakness	Gallbladder	Sensory neuropathy with upper motor neuron dysfunction	ANNA1	5 months	Died, and no immunotherapy was initiated
67/F (33)	Facial and cervical erythema accompanied by pruritus and arthralgia	Gallbladder	Dermatomyositis	Not tested	2 months	Improved with tumor resection
72/F (34)	Sudden onset of vertigo followed by impaired consciousness	Gallbladder	Opsoclonus-Myoclonus Syndrome	Negative	Not mentioned	Died despite immunotherapy
81/F (35)	Involuntary left limb movement	Gallbladder	Chorea	Negative	Not mentioned	Improved with immunotherapy and tumor resection
70/M (36)	Dysarthria, spasticity, ataxia	Gallbladder	Cerebellar degeneration	PCA-1	Not mentioned	Died despite immunotherapy

AMPH, anti-amphiphysin, GAD65, Glutamic acid decarboxylase-6; P/Q-type, P/Q-type voltage-gated calcium channel; CASPR2, Contactin-associated protein-like 2; GM,: ganglioside M1; PCA-1, Purkinje cell cytoplasmic antibody type I.. *Tumor diagnosed prior to PNS onset. Other studies have also reported Ma2 IgG, KLHL11 IgG or MAP1B IgG in association with lung adenocarcinoma, but details of the clinical presentation of individual patients were not reported (12, 15, 17).

SKOR2 is a nuclear oncoprotein belonging to the Sloan-Kettering Virus family. Its expression is restricted to the human CNS tissue, mainly the cerebellum, mesencephalon, and diencephalon, with some expression in the hippocampus and cortex (37, 38). In the cerebellum, it is primarily expressed in post-mitotic Purkinje cell nuclei (38). The function of the SKOR2 protein is not fully understood; however, it is thought to repress the transcriptional activity of Smad proteins. Smad proteins are the main signal transducers for transforming growth factor beta (TGF-B) receptors. Therefore, SKOR2 causes inhibition of TGF-B signaling (37, 39). Studies have also shown that the SKOR2 gene is essential for cerebellar development, specifically the embryonic migration of Purkinje and granule cells and their intercellular signaling in adults (40, 41). Since SKOR2 is a nuclear protein, it is unlikely that SKOR2 IgG plays a role in the pathogenesis of PNS. SKOR2 autoantibodies may serve as a marker of a cytotoxic T-cell–mediated injury directed through T-cell receptors recognizing SKOR2 peptides, similar to PCA1 or KLHL11 autoantibody-associated paraneoplastic cerebellar degeneration (12, 42). This is further supported by the poor long-term outcomes noted in these cases, similar to other cytotoxic T-cell-mediated paraneoplastic syndromes (11, 12, 42).

One of the limitations of this study is the description of only two SKOR2 IgG seropositive cases. Additionally, even though both cases had progressive neurologic presentations in the setting of underlying cancer, consistent with other PNS presentations, their phenotypes were variable. Due to the lack of access to gallbladder adenocarcinoma tissue in the second case, we were not able to demonstrate SKOR2 expression in the tumor. These limitations highlight the importance of detecting additional SKOR2 IgG-positive cases using methods such as tissue IFA and CBA, which will aid in better characterization of the clinical phenotype and risk of cancer association. If additional cases continue to support a >70% frequency of underlying malignancy in SKOR-2 IgG seropositive cases, this antibody could be characterized as a high-risk paraneoplastic antibody (1).

## Conclusions

SKOR2 IgG represents a novel biomarker for a PNS associated with lung or gallbladder adenocarcinoma. Future studies evaluating larger SKOR2 IgG seropositive cohorts will help to better categorize the associated neurological phenotype and risk of underlying malignancy.

## Data availability statement

Study data (raw sequence reads) was deposited in Sequence Read Archive (SRA) under BioProject accession PRJNA1004422.

## Ethics statement

The studies involving humans was approved by Mayo Clinic, Rochester, Minnesota (IRB#08-006647). The studies was conducted in accordance with the local legislation and institutional requirements. This research was carried out utilizing residual serum/CSF samples received at Mayo Clinic Neuroimmunology laboratory for clinical neural autoantibody evaluation. Clinical information was collected from the managing physician for the purposes of autoantibody development or validation. The study carried out for identification and confirmation of the putative autoantibody was covered by IRB#08-006647, in compliance with the applicable regulations for the protection of human subjects and with Mayo Clinic Institutional Policies.

## Author contributions

Study conception and design: MR and DD. Data acquisition and analysis: All authors. Drafting of the manuscript or figures: MR, AMK and DD. Study supervision: DD. All authors contributed to the article and approved the submitted version.

## Supplementary material

Supplementary Table This file contains raw counts and enrichment scores of all oligos observed in both cases (case 1 CSF in and case 2 serum in ). Each case and its corresponding set of controls were processed independently. The raw read counts matching each oligo are shown. An enrichment score for each oligo observed in a case was computed by dividing its corresponding read counts by the average read counts of the same oligo observed in the controls. Oligos were sorted based on enrichment scores. Of all the proteins that ranked high (i.e., the top 20), SKOR2 was present in both cases.
